# Application of the New Irrigation Protocol to Reduce Recurrence Rate in the Management Of Periprosthetic Joint Infection

**DOI:** 10.1111/os.13948

**Published:** 2024-01-18

**Authors:** Xiaoyu Wu, Weishen Chen, Rong Rong, Baiqi Pan, Xuantao Hu, Linli Zheng, Aerman Alimu, Chenghan Chu, Yucheng Tu, Ziji Zhang, Yongyu Ye, Minghui Gu, Puyi Sheng

**Affiliations:** ^1^ Department of Joint Surgery The First Affiliated Hospital of Sun Yat‐Sen University Guangzhou China; ^2^ Guangdong Provincial Key Laboratory of Orthopaedics and Traumatology The First Affiliated Hospital of Sun Yat‐sen University Guangzhou China; ^3^ Department of Nosocomial Infection The First Affiliated Hospital of Sun Yat‐Sen University Guangzhou China; ^4^ Department of Spinal Surgery Guangdong Provincial People's Hospital Guangzhou China

**Keywords:** Irrigation protocol, Periprosthetic joint infection, Recurrence, Revision, *Staphylococcus aureus*

## Abstract

**Objective:**

Irrigation is a conventional treatment for acute and chronic periprosthetic joint infections (PJI). However, there has been no unified standard for irrigation during surgery for PJI in the past, and the efficacy is uncertain. The purpose of this study is to create a new irrigation protocol to enhance the infection control rate and reduce the postoperative recurrence rate of PJI patients.

**Methods:**

We conducted a single‐institution retrospective review with a total of 56 patients who underwent revision total hip or knee arthroplasties due to PJI from January 2011 to January 2022. Conventional irrigation (CI) was used in 32 cases, and standard operating procedure of irrigation (SOPI) was used in 24. The CI protocol carries out an empirical irrigation after debridement, which is quite random. Our SOPI protocol clearly stipulates the soaking concentration and time of hydrogen peroxide and povidone‐iodine. The irrigation is carried out three times, and tissue samples are taken from multiple parts before and after irrigation, which are sent for microbial culture. The important statistical indicators were the rate of positive microbiological culture and postoperative recurrence rate with an average follow‐up of 24 average months.

**Results:**

The drainage volume was lower in the SOPI group than in the CI group on postoperative day 3 (*p* < 0.01) and 7 (*p* = 0.016). In addition, the percentage of positive microbiological cultures after the third irrigation was less than that before (*p* < 0.01) and after (*p* < 0.01) the first irrigation. The most common causative organism was *Staphylococcus aureus*, which was detected in 25.0% and 12.5% of the SOPI and CI groups, respectively. The failure rate at the final follow‐up was 8.3% and 31.3% (*p* = 0.039) for the SOPI and CI groups, respectively.

**Conclusion:**

Compared with the traditional CI method, SOPI standardized the soaking time of hydrogen peroxide and povidone‐iodine, increased the frequency of and irrigation, and proved that microorganisms were almost completely removed through the microbial culture of multiple tissues. SOPI has the potential to become a standardized irrigation process worthy of promotion, effectively reducing the postoperative recurrence rate of PJI patients.

## Graphical abstract

The recurrence rate of SOPI group was significantly lower than that of CI group.

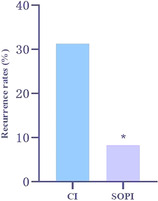

## Introduction

Periprosthetic joint infection (PJI) is a rare but catastrophic complication of total joint arthroplasty (TJA) and partial joint arthroplasty (PJA).^1^, ^2^ About 1% of patients undergoing knee arthroplasties and 1%–2% of patients undergoing hip arthroplasties have PJI.^3^, ^4^ The occurrence reason of PJI was mainly produced by the invasion of pathogenic microorganisms, including bacteria and fungi. PJI can be diagnosed by the sinus tract or persistent wound drainage over a joint prosthesis and positive for microorganism culture.^5^ In addition, various synovial fluid and blood indices, imaging examinations and metagenome sequencing also have great significance for the diagnosis.^6^ The bacterial biofilm attached to the prosthesis is one of the important reasons for the formation of chronic infections due to its resistance to antibiotics, immune system, and mechanical debridement.^7^ Some immunosuppressive factors (such as PD‐L1) may play an important role in the formation of the immunosuppressive microenvironment in the joint capsule.^8^ In addition, multiple research has found the microenvironment features mainly characterized by inhibitory immune cell infiltration in PJI.^9^, ^10^ The above reasons cause the difficulty of treatment of PJI. PJI is mainly treated by surgery, which includes two‐stage revision arthroplasty or debridement, antibiotics, and implant retention (DAIR). The surgical operative methods were determined by the degree, time and even the specific location of the infection.^11^, ^12^ DAIR showed a good success rate in cases of early acute infection by multidrug‐sensitive bacteria, but it is not suitable for patients with bacterial resistance.^13^ Immunocompetent patients with healthy soft tissues, only mild or moderate bone loss, confirmed infection microorganisms and its sensitivity, can be treated with one‐stage revision.^14^ Two‐stage revision remains the gold standard for the treatment of PJI.^15^ Even if therapied by operation, there were still more than 15% of patients require re‐revision after 10 years.^16^ It is important to find new ideas in the existing treatment methods of PJI to reduce the postoperative recurrence rate. Previous studies have noted that thorough irrigation is a critical step in acute and chronic PJI surgery and plays an important role in eradicating pathogenic microorganisms.^17^, ^18^

Initially, irrigation was generally performed with normal saline. With advances in medical treatment, multiple additives have been introduced in irrigation protocols.^19^ These additives are mainly classified into three categories: surfactants, antiseptics, and antibiotics.^20^ The International Consensus on Orthopedic Infections recommends the use of antibiotic irrigation for PJI.^21^ Antiseptics, including diluted povidone‐iodine, hydrogen peroxide, and acetic acid, are the most widely used irrigation solutions.^22^ However, there are no clear guidelines for the optimal irrigation protocol, amount, and time of operation. Most studies used diluted povidone‐iodine and hydrogen peroxide as irrigation solutions.^23^, ^24^ However, this approach has limited effectiveness, as previously shown in our institution, as some patients experienced persistent pain or recurrence.

Based upon the conditions described above, we have proposed two scientific points: (i) the recurrence of PJI may be related to the residual pathogenic microorganisms caused by incomplete intraoperative irrigation; and (ii) the removal of pathogenic microorganisms depends on appropriate soaking time and irrigation times. Our research aims to promote a standardized irrigation process: finding the most suitable soaking time and irrigation frequency. We validated the scavenging effect of pathogenic microorganisms through multiple times and sites of microbiological culture, and ultimately demonstrated the effectiveness of the new protocol by calculating the recurrence rate. Therefore, we formulated a standard operating procedure of irrigation (SOPI) protocol to eradicate pathogenic microorganisms more effectively and improve clinical outcomes. We compared patients in the SOPI and conventional irrigation (CI) groups to explore whether the new irrigation protocol reduced the postoperative recurrence rate. We estimate that the SOPI protocol can more effectively eliminate pathogenic microorganisms, thereby reducing the recurrence rate of patients.

## Methods

### Demographic Characteristics and Management

We conducted a single‐center retrospective cohort study to compare the differences between the SOPI and CI groups. All patients with PJI who underwent revision total joint arthroplasty between 2011 and 2022 were identified. Patients with PJI were confirmed according to the diagnostic criteria of the Musculoskeletal Infection Society (MSIS).^25^ All cases were did not suffer from immune diseases and were without a history of revision surgeries. Long‐term follow‐up data were available for all patients. This study is the first to standardize the irrigation protocol and has obtained institutional review board and ethics committee approval (IRB number [2021]676) from our hospital.

Physical examination, history taking, and preoperative assessment were performed for all patients. Preoperative examinations included laboratory examination, radiography, CT, and MRI. The patients underwent surgery with proper management of blood pressure and blood glucose levels. Vancomycin and rifampicin were used to treat PJI postoperatively, and the administration of antibiotics was adjusted according to the outcome of the microbiological culture. Heparin or factor Xa inhibitor was administered 24 h after the operation to prevent deep venous thrombosis. The medial parapatellar approach was applied in revision total knee arthroplasty, and the posterior lateral approach was applied in revision hip arthroplasty. All operations were performed by one of four experienced arthroplasty surgeons in our hospital.

### Surgical Management

Forty‐five patients included in the study admitted for treatment were diagnosed with chronic PJI, which were treated with two‐stage arthroplasty. Four patients diagnosed with acute PJI were treated with DAIR, and seven patients were treated with one stage revision arthroplasty. Therefore, they were not sub‐grouped for further analysis. The joint fluid and tissues obtained from joint cavity puncture from all patients were used for microbiological culture. Methods combined spinal‐epidural anesthesia (CSEA) and general anesthesia were operated. Median tourniquet time overall was 60 min. Intravenous infusion of tranexamic acid (20mg/kg) was given 5–10 min before skin incision. The prosthesis of antibiotic‐loaded acrylic cement (Smith & Nephew, USA) was implanted in the primary operation of two‐stage arthroplasty. After implantation, it was slightly pressurized and maintained until the bone cement solidified. This method refers to the 2018 PJI Philadelphia International Consensus. The bone cement augmented screw fixation was performed when bone defect encountered during surgery. For serious cases, endoprosthesis with longer shaft should be utilized to improve the stability. When the prosthesis was difficult to remove, we further cleaned the scar tissue, prominent bone tissue and bone cement.

### Postoperative Rehabilitation

The patients were treated with vancomycin (0.5–1 g, iv, q12 h) and rifampicin (450 mg, po, qd) for 2 weeks. Rifampicin and oral sensitive antibiotics were continued for 3 months after discharge. The patient's weight and muscle function recovered about 1 month after discharge. The postoperative knee flexion angle of the patients recovered to 90–120°. The X‐ray image showed good positioning of the prosthesis with infection significantly cleared.

### Irrigation Protocol

The SOPI for patients with PJI was initiated at our institution in January 2017. Patients with one‐or two‐stage revision arthroplasty or DAIR were included in our study (Table 1).The detailed procedure was as follows: (i) resect the infected and necrotic tissue and completely remove the prosthesis and prosthetic membrane between the prosthesis and the bone (except DAIR); (ii) infuse the incision, articular cavity, and medullary cavity with H_2_O_2_ for 10 min; (iii) irrigate with 1.5 L of normal saline; (iv) immerse in diluted povidone‐iodine for 10 min; (v) irrigate again with 1.5 L of normal saline; (vi) obtain culture from multiple sites including synovial fluid (hip and knee) and tissues surrounding the acetabulum (hip), femur (hip and knee), tibia (knee), patella (knee), sinus tract (hip and knee); and (vii) repeat the procedure three times, using sterile surgical draping, instruments, gowns, and gloves after the last irrigation. The control cohort was selected from patients with PJI using the CI protocol at our hospital. The irrigation solution of CI protocol was also povidone‐iodine and H_2_O_2_. However, there is no unified standard for the irrigation and soaking time, which only depends on experience. The following situations were considered as recurrence: (i) healed wound with fistula, drainage, or excessive pain; (ii) recurrence of infection caused by the same microorganism; (iii) reoperation due to infection; and (iv) occurrence of PJI‐related mortality.

**TABLE 1:** Operative methods used for patients in the SOPI and CI groups.

Surgical method	CI (*n* = 32)	SOPI (*n* = 24)
One‐stage revision arthroplasty	6	1
Two‐stage revision arthroplasty	23	22
DAIR	3	1

Abbreviations: CI, conventional irrigation; DAIR, debridement, antibiotics, and implant retention; SOPI, standard operating procedeure of irrigation.

### Outcome Measures

We calculated the preoperative demographic data and clinical features of patients, including gender, age, body mass index (BMI), postoperative complications, joint function score, and the correlation indicators including drainage volume, temperature, C‐reactive protein (CRP), white blood cell (WBC) count, neutrophil count (NEUT), lymphocyte count (LY), and NEUT/LY on the 1st, 3rd, and 7th postoperative days. The follow‐up results were determined as recurrence: (i) admission due to recurrent infection; (ii) pus exudation from the incision; and (iii) persistent pain that cannot be relieved.

### Statistical Analysis

Measurement data (Age, BMI, Joint function score, Correlation indicators) were analyzed using the independent sample *t*‐test, and enumeration data (sex, position, postoperative complications, positive rate and recurrence rate) were analyzed using the chi‐square test. All normally distributed data are presented as mean ± standard deviation. All statistical analyses were performed using SPSS 22.0 (IBM Corporation, Armonk, NY, USA). Statistical significance was set at *p* < 0.05.

## Results

### Demographic Data and Clinical Features of SOPI and CI Groups

The demographic and clinical characteristics of the enrolled patients in the SOPI (*n* = 24) and CI (*n* = 32) groups are shown in Table 2. There were no statistically significant differences in age, sex, classification, antibiotic use time, incision effusion, periprosthetic loosening, periprosthetic fracture, hospital for special surgery (HSS) score (knee) and Harris score (Hip) between the two groups (*p* > 0.05). We always monitored the indicators of liver function during the application of antibiotics, and did not find significant changes.

**TABLE 2:** Demographic data and clinical features of the patients in the SOPI and CI groups.

Demographic data and clinical features	CI (*n* = 32)	SOPI (*n* = 24)	*p*
Age (years)	67.47 ± 11.76	64.50 ± 12.84	0.379*
Sex			0.530^†^
Male	12	11	
Female	20	13	
Joint			0.352^†^
Hip	16	15	
Knee	16	9	
BMI (kg/m^2^)	24.63 ± 4.13	23.26 ± 3.45	0.194*
Postoperative complications			
Incision effusion	5	2	0.686^†^
Periprosthetic loosening	2	0	0.501^†^
Periprosthetic fracture	0	0	>0.99^†^
Joint function score			
HSS score (knee)	82.25 ± 9.54	88.22 ± 7.98	0.126*
Harris score (Hip)	79.44 ± 9.54	78.33 ± 8.23	0.733*

* *p*‐value was calculated by the sample *t*‐test.

^†^ *p*‐value was calculated by the *χ* ^2^‐test. *p* < 0.05 was regarded as statistically significant.

### Correlation Indicators at Different Time Points of PJI

Univariate analysis was used to compare differences in laboratory‐related indicators and drainage volume between the SOPI and CI groups. There was a significant difference in drainage volume on days 3 and 7 between the SOPI and CI groups (*p* < 0.05). The differences in temperature, CRP, WBC count, NEUT, LY, and NEUT/LY between the SOPI and CI groups were not significant (*p* > 0.05) (Table 3).

**TABLE 3:** Correlation indicators at different time points of PJI.

Correlation indicators	Detection	CI (*n* = 32)	SOPI (*n* = 24)	*p*
Drainage volume (mL)	a	281.79 ± 196.68	194.32 ± 178.82	0.093
	b	111.35 ± 88.98	54.63 ± 43.32	0.003
	c	12.50 ± 34.30	6.99 ± 12.32	0.016
Temperature (°C)	d	36.56 ± 0.23	36.72 ± 0.46	0.102
	a	36.79 ± 0.40	37.05 ± 0.67	0.073
	e	36.77 ± 0.31	36.58 ± 0.33	0.036
CRP (mg/L)	d	30.43 ± 27.77	53.50 ± 61.46	0.097
	a	53.96 ± 29.12	73.76 ± 48.27	0.083
	e	20.57 ± 14.91	23.15 ± 23.95	0.646
WBC (×10^9^/L)	d	7.10 ± 2.36	8.28 ± 2.56	0.078
	a	8.70 ± 2.70	9.81 ± 2.22	0.104
	e	6.53 ± 1.95	6.83 ± 2.46	0.613
NEUT (×10^9^/L)	d	4.61 ± 2.05	5.43 ± 2.30	0.164
	a	6.89 ± 2.59	7.98 ± 1.94	0.089
	e	3.81 ± 1.47	4.19 ± 1.79	0.382
LY (×10^9^/L)	d	1.73 ± 0.73	1.70 ± 0.81	0.912
	a	1.19 ± 0.42	1.07 ± 0.46	0.326
	e	1.62 ± 0.85	1.52 ± 0.57	0.602
NEUT/LY	d	3.41 ± 2.89	4.22 ± 3.15	0.322
	a	7.78 ± 7.70	9.70 ± 6.18	0.319
	e	3.06 ± 1.82	2.88 ± 0.97	0.655

Abbreviations: CI, conventional irrigation; CRP, C‐reactive protein; LY, lymphocyte count; NEUT, neutrophil count; PJI, periprosthetic joint infection; SOPI, standard operating procedure of irrigation; WBC, white blood cell count.

*Notes*: a, first postoperative day; b, third postoperative day; c, seventh postoperative day; d, admission day; e, discharge day. *p*‐value was calculated by the sample *t*‐test. *p* < 0.05 was regarded as statistically significant.

### Types of Pathogenic Microorganisms of SOPI and CI Groups

**Fig. 1:**
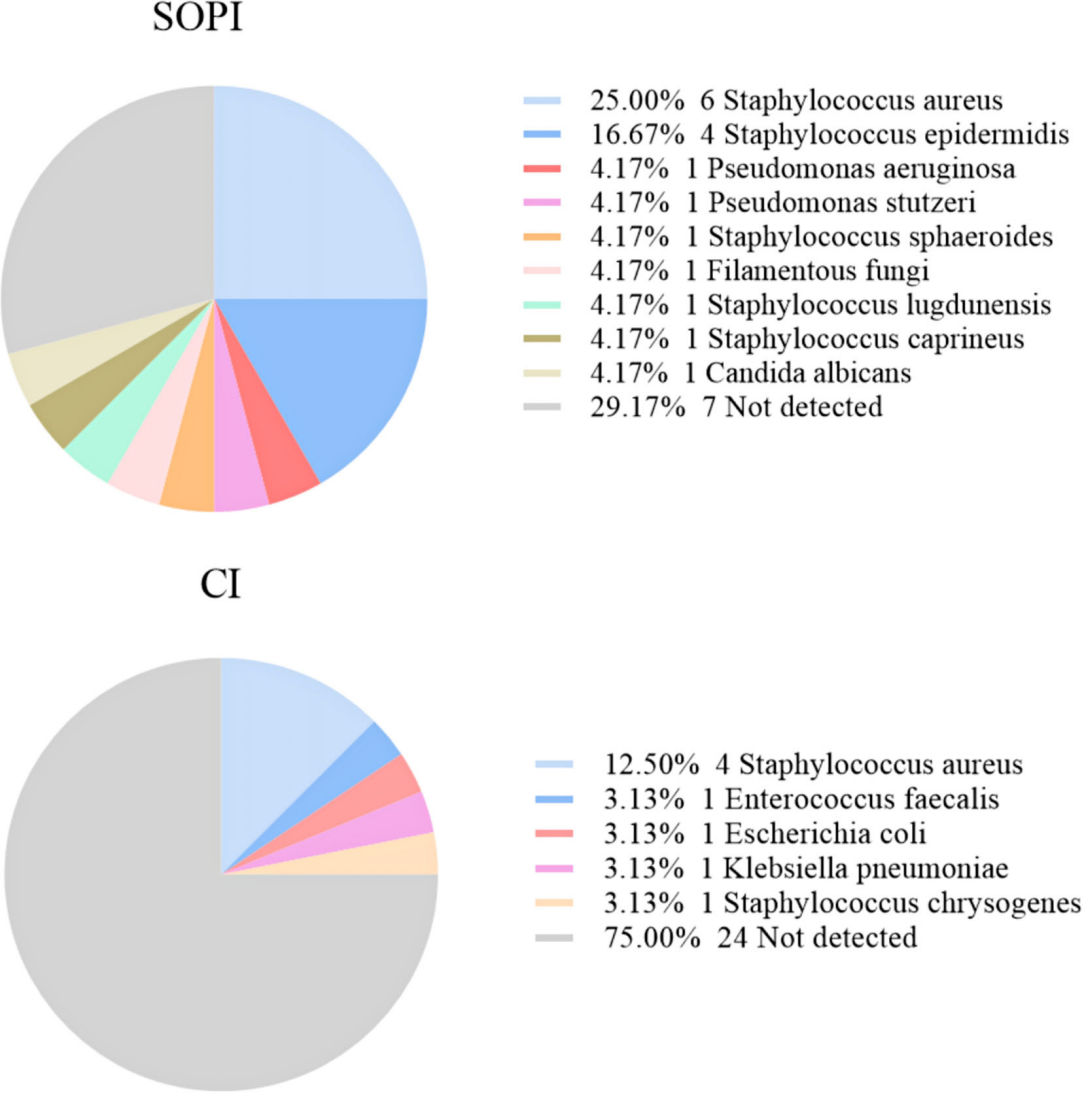
Types of pathogenic microorganisms from standard operating procedure of irrigation (SOPI) and conventional irrigation (CI) groups. *Staphylococcus aureus* was the most common pathogenic microorganism of periprosthetic joint infections (PJI).

The most common causative organism was *Staphylococcus aureus*, which was detected in 25.0% and 12.5% of the SOPI and CI groups, respectively (Figure 1).

### Positive Rate of Microbiological Culture in SOPI Group

**Fig. 2:**
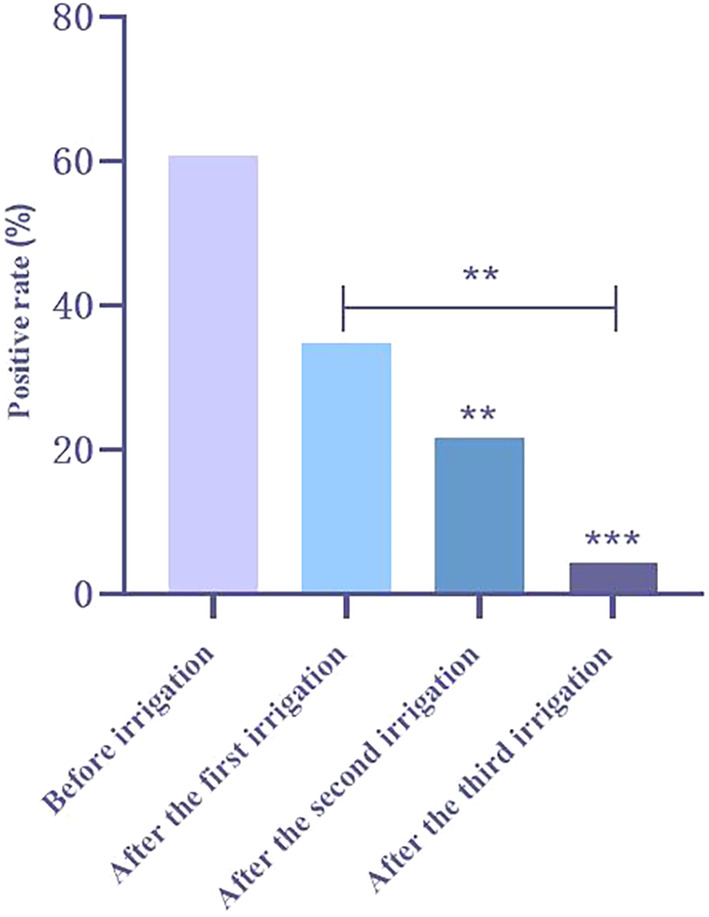
Positive rate of microbiological culture in standard operating procedure of irrigation (SOPI) group. A *χ* ^2^‐test was applied to calculate the proportional difference. **p < 0.01, ***p < 0.001.

SOPI patients were divided into four groups according to irrigation frequency: before irrigation, after the first irrigation, after the second irrigation, and after the third irrigation. One patient was excluded because of sample contamination due to an operational error. Microbiological cultures were performed on the samples before and after each irrigation. Positive microbiological cultures decreased after the second (*p* < 0.05) and third irrigations (*p* < 0.05) compared with before irrigation. More importantly, it decreased after the third irrigation compared with that after the first irrigation (*p* < 0.05) (Figure 2).

**Fig. 3:**
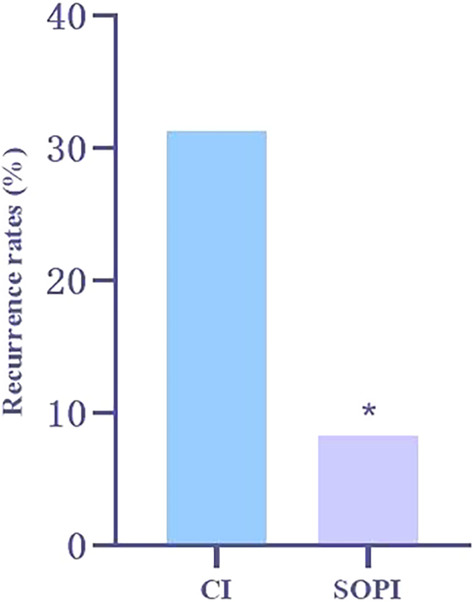
Recurrence rate of periprosthetic joint infections (PJI) between standard operating procedure of irrigation (SOPI) and conventional irrigation (CI) groups. A *χ* ^2^‐test was applied to calculate the proportional difference. *p < 0.05.

All patients were followed up for 24.1 ± 7.4 months. The shortest follow‐up time was 12 months. There were 2/24 recurrences in the SOPI group (8.33%) and 10/32 in the CI group (31.25%). This difference was statistically significant (*p* = 0.039) (Figure 3).

## Discussion

In an effort to improve the success of PJI, the authors of this study implemented a protocol with new irrigation method. To our knowledge, no study has reported on the effect of irrigation in the setting of established PJI. In looking at further adjuncts to maximize the success of PJI treatment, the authors of this study decided to increase soaking time and irrigation. The results showed that compared with the CI group, our SOPI group showed a significant decrease in drainage volume at 7 and 14 days after surgery, although there was no statistically significant difference in postoperative temperature, CRP, WBC, NEUT, LY, and NEUT/LY. Moreover, the positive rate of microbial cultivation significantly decreased after completing three rounds of irrigation. SOPI protocol successfully reduced the recurrence rate of PJI.

### Irrigation Fluid and Irrigation Time

Several studies have been conducted on the application of irrigation solutions. Researchers have applied various types of irrigation solutions, such as surfactants, antibiotics, and antiseptics to PJI surgery, where they have different efficacies.^22^, ^26^ The ideal irrigation solution should establish the minimum biofilm eradication concentration with a bacterial load reduction of at least 99.9%.^27^ However, little research has been conducted on irrigation protocols. We believe standardizing the irrigation process can maximize the effects of the irrigation solution.

Povidone‐iodine was also included in multiple protocols for the surgical treatment of PJI.^19^, ^28^ Zubko and Zubko found that hydrogen peroxide and povidone‐iodine were bacteriostatic when used separately and bactericidal when used in conjunction.^29^ We selected a conventional irrigation solution that included povidone‐iodine, hydrogen peroxide, and normal saline in the SOPI protocol. Normal saline (NS) is the most commonly used irrigation solution for debridement. Some studies have demonstrated that diluted povidone‐iodine was significantly better than saline solution irrigation for preventing PJI.^28^ However, the optimal povidone‐iodine dilution has yet to be determined. Povidone‐iodine is effective, with minimal damage to the host tissue at lower concentrations.^24^ Hydrogen peroxide has been proven effective against bacteria, especially gram‐positive organisms, and does not adversely affect the strength of bone cement or metal implants.^30^ Hydrogen peroxide reduces bacterial biofilms.^30^, ^31^ However, the use of hydrogen peroxide can increase the risk of air embolism;^32^ thus, thorough irrigation with normal saline is necessary to prevent such complications. There is little evidence for beneficial results when adding antibiotics to the irrigation solution, and it is not currently recommended by the World Health Organization.^33^, ^34^

We effectively decreased the prevalence of pathogenic microorganisms by increasing irrigation time and frequency. Usually, the wound is irrigated with diluted povidone‐iodine for 3 min;^19^, ^28^ however, we extended the irrigation time to 10 min. We applied hydrogen peroxide followed by a dilute povidone‐iodine solution, after which we pulsed irrigation with 1500 mL of normal saline. Poilvache *et al*. demonstrated that pulsed irrigation can reduce the bacterial load and remove most of the biofilm.^35^ We used a pulsed irrigation device that projected normal saline intermittently at pressures between 30 and 350 kPa. Most studies performed irrigation 1–2 times and did not mention when tissues were taken for microbiological culture.^24^ We performed irrigation three times and sent the tissues for microbiological culture before the first irrigation and after each irrigation.

### Multiple Times and Stites of Microbiological Culture

No research have ever collected specimens separately to detect pathogenic microorganisms after multiple standard irrigation procedures. At present, there is no unified standard for intraoperative irrigation, so it is difficult to know what degree of irrigation can completely remove microorganisms. Although there is much research on irrigation fluid, it seems that no researcher has paid attention to the methodology of the whole irrigation process. We developed an optimal process to remove pathogenic bacteria through intraoperative multi‐site and multi‐step microbial detection, and its effectiveness has been proved. Through multiple irrigation, we effectively reduced the percentage of positive microbiological cultures from 34.8% to 4.3% after the third irrigation. This finding is of great significance for the eradication of pathogenic microorganisms. In addition, the rate of positive microbiological culture results in our study was low, and the most likely reason was the application of antibiotics before the operation. In chronic PJI, most pathogenic microorganisms exist in the biofilm and attach to the prosthesis surface surrounding the tissues. However, the culture of pathogenic microorganisms depends on separate floating particles, which also contributes to the low detection rate. Our results showed that the rate of positive microbiological culture in the SOPI group was significantly higher than that in the CI group, indicating that microbiological culture in multiple parts of the surgical site was more effective for identifying pathogens. Therefore, we recommend taking tissue samples from multiple sites for microbiological culture in PJI surgery to avoid false negatives.

### Reduction of Recurrence Rate

We implemented SOPI to remove pathogenic microorganisms successfully to the maximum extent through standardized debridement and irrigation procedures. However, our ultimate aim is to improve the prognosis and survival quality of patients, and reduce the recurrence rate. It was found that the possibility of permanent eradication of infection in PJI reoperation was less than 50% through long‐term follow‐up.^36^, ^37^ Royo *et al*. applied dilute povidone‐iodine and hydrogen peroxide for irrigation, with a success rate of 73.5%.^38^ Riesgo *et al*. applied the vancomycin povidone‐iodine protocol, which improved the success rate from 63% to 83.3%.^39^ Obviously, the postoperative recurrence rate remains high, although different irrigation solution were applied during PJI operation. The results of our matched cohort study demonstrated that the implementation of SOPI increased the PJI success rate at our institution from 68.75% to 91.30%. This improvement was significant when using the SOPI protocol. In addition, the SOPI protocol provided the most conventional irrigation solution, including povidone‐iodine and hydrogen peroxide. There was no significant difference in various postoperative complications and liver function between the two groups. It can be considered that the SOPI protocol is a very safe and reliable procedure. All patients diagnosed with PJI in our hospital were treated with antibiotics to limit risk of infection before operation. Although implant prostheses are different, we consider that this does not affect infection clearance. These factors did not affect the reliability and rationality of valuation conclusions. We suggested that thorough debridement and a standardized irrigation protocol with irrigation solutions of povidone‐iodine, hydrogen peroxide, and normal saline would minimize the difficulty in treating PJI.

### Limitations

Our study refined and standardized conventional irrigation solutions, and the findings were validated regarding the rate of positive microbiological cultures and follow‐up recurrence rates. There are some limitations beyond those inherent in retrospective studies. The sample sizes were relatively small, with only 24 cases in the SOPI group. The duration of follow‐up was short, with a minimum follow‐up of 3 months. The different pathogenic microorganisms and virulence could also contribute to the different incidences of recurrence, and these variables were not controlled. Thus, a longer follow‐up period with a larger number of cases is needed to validate our protocol.

## Conclusion

The SOPI protocol is safe and effective in improving the success of PJI treatment compared to CI. It effectively reduced the percentage of positive microbiological cultures and the postoperative recurrence rate.

## Conflict of Interest Statement

The authors declare that the research was conducted in the absence of any commercial or financial relationships that could be construed as a potential conflict of interest.

## Ethics Statement

All methods were carried out in accordance with relevant guidelines and regulations. This study was approved by the institutional review board and ethics committee of our hospital. All human subjects signed informed consent in the study.

## Author Contributions

Puyi Sheng and Xiaoyu Wu conceived the research topic; Xiaoyu Wu, Ziji Zhang, and Minghui Gu collected the raw data from clinical record. Xiaoyu Wu constructed the database files and drafted the manuscript together with Weishen Chen and Rong Rong. Ziji Zhang and Minghui Gu provide writing assistance. Xiaoyu Wu, Rong Rong, Baiqi Pan, Xuantao Hu, Linli Zheng, Aerman Alimu, Chenghan Chu, and Yucheng Tu performed proof reading of the article.

## Funding Information

This study was supported by the National Natural Science Foundation of China (Grant number 81972050 and 82172405), Guangdong Basic and Applied Basic Research Foundation (Grant number 2023A1515030030), Science and Technology Projects in Guangzhou (Grant number 2023A04J2187). No benefits in any form have been received or will be received from a commercial party related directly or indirectly to the subject of this article.

## Data Availability

The original contributions presented in the study are included in the article, further inquiries can be directed to the corresponding authors.
